# Multidisciplinary Investigations on *Galphimia glauca*: A Mexican Medicinal Plant with Pharmacological Potential

**DOI:** 10.3390/molecules23112985

**Published:** 2018-11-15

**Authors:** Ashutosh Sharma, Paola Isabel Angulo-Bejarano, Alfredo Madariaga-Navarrete, Goldie Oza, Hafiz M. N. Iqbal, Alexandre Cardoso-Taketa, Maria Luisa Villarreal

**Affiliations:** 1 Tecnologico de Monterrey, School of Engineering and Sciences, Campus Queretaro, Av. Epigmenio González No. 500, Fracc. San Pablo, Queretaro CP 76130, Mexico; pangulobe@tec.mx; 2 Área Académica de Ciencias Agrícolas y Forestales, Instituto de Ciencias Agropecuarias, Universidad Autónoma del Estado de Hidalgo, Tulancingo CP 42000, Mexico; alfredomadariaga60@gmail.com; 3 Centro de Investigación y Desarrollo Tecnológico en Electroquímica (CIDETEQ), Parque Tecnológico, Querétaro S/N, Sanfandila. Pedro Escobedo, Querétaro CP 76703, Mexico; goza@cideteq.mx; 4 Tecnologico de Monterrey, School of Engineering and Sciences, Campus Monterrey, Ave. Eugenio Garza Sada 2501, Monterrey CP 64849, Mexico; hafiz.iqbal@itesm.mx; 5 Centro de Investigación en Biotecnología (CEIB), Universidad Autónoma del Estado de Morelos (UAEM), Cuernavaca CP 62209, Mexico; ataketa@uaem.mx (A.C.-T.); luisav@uaem.mx (M.L.V.)

**Keywords:** *Galphimia glauca*, phytochemicals, anxiety, biotechnology

## Abstract

*Galphimia glauca* (Cav.) Kuntze is an important endemic plant species, which possesses many medicinal properties and has been used in the Mexican traditional medicine for its sedative, anxiolytic, anticonvulsant, antiasthmatic and antiallergic properties. The therapeutic properties of this plant are mainly due to the presence of diverse bioactive compounds such as flavonoids, triterpenoids, and phenolics. Several triterpenoids and flavonoids compounds have been isolated and identified. Modern studies have demonstrated many biological activities such as anti-inflammatory, antidiarrheal, gastroenteritis, antimalarial and cytotoxic activities. Nevertheless, many studies are restricted to the crude extract, and many bioactive compounds are yet to be identified and validated according to its traditional use. However, its commercial exploitation and use are highly limited due to the non-availability of enough plant material and lack of knowledge about its agronomical practices. Moreover, the misinterpretation and mislabeling of closely related species of the genus *Galphimia Cav.* as *G. glauca or G. gracilis* is a common problem for its rigorous scientific study and commercial exploitation. The present review provides comprehensive knowledge based on the available scientific literature. To the best of our knowledge, this is the first review on *G. glauca*. This comprehensive information will certainly provide a guide for the better understanding and utilization of *G. glauca* for its scientific and industrial exploitation.

## 1. Introduction

*Galphimia glauca* Cav. also known as “*calderona amarilla*”, “*flor estrella*”, “*hierba del desprecio*” “*hierba del cuervo*”, “*ojo de gallina*”, among other names, is an important Mexican plant species for the treatment of anxiety and depression among other ailments since pre-Hispanic times [1,2]. Its distribution is limited to only a few states in Mexico [3], and only two main zones generate cultivars that are associated with the highest concentrations of its main anxiolytic compound: galphimine B [4]. Besides its use for its effects on the central nervous system (CNS) [5], it has also been employed for the treatment of asthma, allergies, diarrhea, gastroenteritis, and malaria, among others [6]. These uses are tightly linked with the presence of phytochemical compounds, mainly a triterpenoid family of nine known compounds named galphimines, isolated for the first time in this plant [4,7]. Also, they contain high levels of antioxidant compounds such as flavonoids, namely quercetin [8]. 

This review intends to provide comprehensive information on *Galphimia glauca*, which includes its botanical, phytochemical, biotechnological aspects and their beneficial effects on human health. In addition, we address the fact that some *Galphimia* spp. are incorrectly identified with different names. The provided data will certainly work as a guide for the researchers which will support adequate scientific and commercial exploitation of this important plant species. This review includes the information collected from various scientific databases such as PubMed, Scopus, EBSCO, Science Direct, and ProQuest. 

Even though *Galphimia glauca* has been intensively studied over the past 25 years, only 43 articles were found to be strictly related with *Galphimia glauca* which were included in this review. Articles other than English language were not considered and several articles or reviews that mention *Galphimia glauca* in studies related with homeopathy or molecular taxonomy of Malpighiaceae family were also excluded. In addition, several research articles book chapters, and reviews related with medicinal plants, nutraceuticals, ethnobotanical uses, and taxonomical features were also revised. Finally, one patent related with the use of galphimine B is also documented in the present review. 

## 2. Botanical Description, Taxonomy, Distribution, and Ecosystems

The Malpighiaceae family includes approximately 71 genera, which spans in 1250 species. The majority of these species are climbers; however, trees and shrubs are also found [9,10]. Plants within the Malpighiaceae family are considered economically important. The Barbados cherry (*Malpighia emarginata*) is high in vitamin C content [11]. Also, hallucinogenic compound production is found in some species of both the *Banisteriopsis* and *Diplopterys* genera [12]. On the other hand, some species of the *Banisteriopsis*, *Malpighia*, *Peixotoa*, *Stigmaphyllon*, and *Galphimia* genera are cultivated as ornamental plants [13]. 

**Figure 1:**
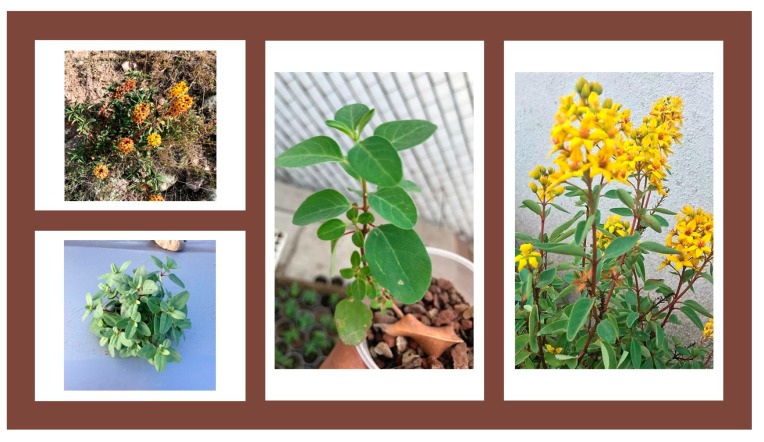
*Galphimia glauca* at different stages of development (photo courtesy of Ashutosh Sharma).

As is the case with many plants belonging to the Malpighiaceae family, most of the species comprised in the *Galphimia* genus are shrubs which can reach up to 4 m, while others form treelets up to 6 m in height. Also, depending on their morphology they can grow as small shrubs up to 1.5 m high [3] (Figure 1). The Malpighiaceae family present high chromosome number variability, and in the case of the Galphimieae tribe, it ranges from 12 to 24 chromosomes, where the last value is the most represented one (62.5%) [14]. 

**Figure 2:**
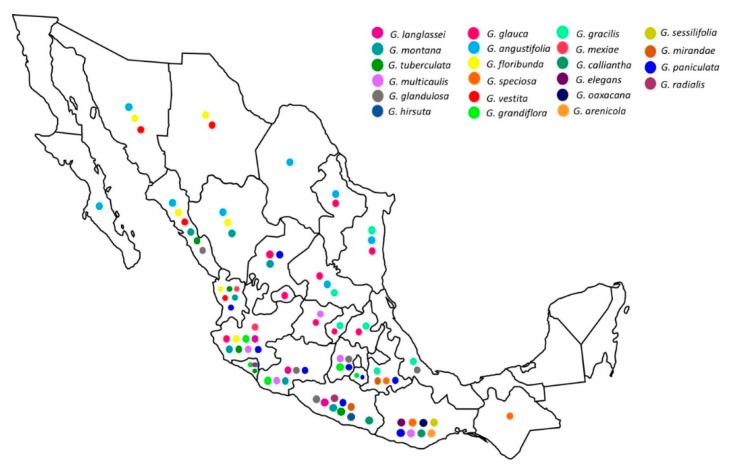
Geographical distribution of the 22 species of the genus *Galphimia*.

The natural distribution of *Galphimia* is mostly in dry habitats. The genus *Galphimia* comprises 26 species, and 22 of these species are concentrated in Mexico; some are considered endemic [3]. In fact, *G*. *speciosa* extends from Mexican territories into Central America, and *G*. *angustifolia* distributes into the United States (Texas). Some Mexican species are found typically in dry environments within pine-oak forests and shrublands, and just one of them (*G*. *grandiflora*) is found in mesic sites in oak, pine and fir forests. Other species can yet be found in really dry environments such as dunes in the Pacific coast of the Tehuantepec Isthmus [3] (Figure 2). 

**Figure 3:**
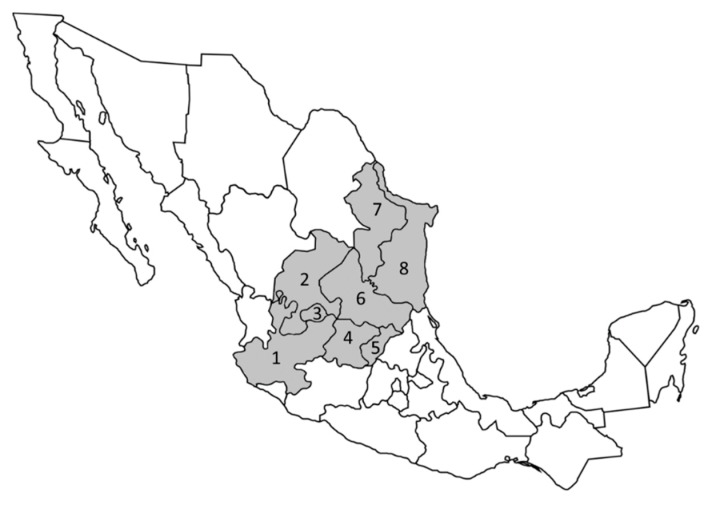
*Galphimia glauca* distribution in Mexico according to Anderson [2]. Gray color indicates the corresponding states. 1. Jalisco; 2. Zacatecas; 3. Aguascalientes; 4. Guanajuato; 5. Queretaro; 6. San Luis Potosi; 7. Nuevo Leon; 8. Tamaulipas.

Interestingly, *Galphimia glauca*, one of the most studied species for its medicinal properties, is not widely represented in Mexico, in fact, its distribution is limited to the following states: Aguascalientes, Guanajuato, Hidalgo, Jalisco, Nuevo Leon, Queretaro, Tamaulipas and Zacatecas (Figure 3). These states comprise part of the central and northeast zones of the country. This distribution pattern can be attributed mainly to their adaptation to the shrubland and pine-oak forest present in these zones [3]. Some species like *Galphimia amambayensis*, *Galphimia australis*, and *Galphimia platyphylla* are found in Paraguay and *Galphimia brasiliensis* found in Brazil [2]. 

Due to the overall aesthetic characteristics of *Galphimia glauca* plants (catchy flower colors) collectors have taken samples and helped in its dispersion outside of its endemic site [3], which makes this plant exotic in other parts of the world. According to certain studies, the presence of *Galphimia glauca* in places such as Cuba and the Virgin Islands has also been reported [15]. In fact, *Galphimia glauca* is also considered as an invasive plant in Cuba [16].

## 3. History and Uses

The use of plants as part of traditional medicine is tightly linked to human civilization development. Different historical documents highlight the use of plants in many cultures [17]. In Mexico, the use of traditional medicine is widely documented. The earliest information is found in “*Libellus de Medicinalibus Indorum Herbis*” or the Codex de la Cruz-Badiano wrote in 1552 which concentrates the information in Aztec medicine available at the time and translated to Latin [18]. This codex includes thorough information as to which plants, their parts and how they were prepared to be used as medicine. Since Mexico is a megadiverse country, the list of plants with potential medical use is large. Many of them have been extensively used since pre-Hispanic times up to our days. Such is the case of plants belonging to the Malpighiaceae family. The use of *Galphimia glauca* in Mexican traditional medicine can be tracked up to the 1500s as described by Hernández [19]. The Aztecs designated this plant as “Totoncapatli” a name formed by “totonqui”: hot, and “patli”: medicine [1]. Nowadays, this plant is also known as “*calderona amarilla*”, “*flor estrella*”, among others [20]. 

The principal uses of this plant are related mainly to its tranquilizing and sedative properties. Historical records include its utilization during certain civil war movements in Mexico, were an infusion was made out of leaves and stems and delivered to soldiers suffering from anxiety episodes [1]. Mexican traditional medicine relates the use of *Galphimia glauca*, to the treatment of “*nervios*” a folk term for an illness whose symptoms are closely related with those of depression and anxiety disorders [20]. This effect is due to various phytochemical compounds that have been identified in this plant species. 

In addition, several studies have described the use of *Galphimia glauca* in different formulations for homeopathic remedies [21]. Thus, in 1985 Wiesenauer and Gaus, [22] described the use of a homeopathic product called Galphimia D6, (a six times potentiation in 90% ethanol), and evaluated its effects in patients suffering from pollinosis. As a result, the use of this D6 formulation was proven to be better than the use of regular *Galphimia glauca* dilutions. Further on, preparations of Galphimia D4 on saccharose globules were studied in patients suffering from pollinosis, the overall effect was a reduction in symptoms versus the placebo population analyzed [23]. Mixtures involving *Galphimia glauca* have also been described. In 2004, a study evaluated the use of a nasal spray containing *Luffa operculata*, *Galphimia glauca*, histamine and sulphur against hay fever symptoms [24]. In brief, the use of this nasal spray was as efficient as the use of commercial cromolyn solution. Homeophatic studies have also been conducted in *Galphimia glauca*-based formulations. Accordingly, the patients experienced several symptoms when using the product such as relaxing, sedative, anxiolytic and antiallergenic effects [25]. 

## 4. Phytochemistry 

*Galphimia glauca* is a rich source of different types of natural products, with a variety of structural patterns. The presence of several of these compounds has been reported in the different plant organs of Mexican “*calderona amarilla*”. 

### 4.1. Phenolic Compounds

The phenolic compounds along with alkaloids and terpenoids constitute one of the most studied groups in phytochemistry [26]. The term “phenolics” comprises a large group of chemical compounds derived from the phenylpropanoid pathway. Their biological activities are highly related with their structure, participating in metabolic and cell signaling pathways and present antioxidant, antiproliferative, pro-apoptotic, anti-angiogenic and anti-inflammatory properties. Along with these properties, they can affect the enzyme and protein function [27]. 

Gallic acid (3,4,5-trihydroxybenzoic acid), one of the simplest phenolic compounds present in plants, is found in grapes, berries, and tea, and it presents antioxidant, anti-inflammatory and anticancer properties [27,28]. The presence of gallic acid, as well as some other complex forms such as tetragalloylquinic acid (1,3,4,5-tetra-*O*-galloylquinic acid), methyl gallate, and ellagic acid, have been reported in *Galphimia glauca* methanolic and ethyl acetate extracts [8,29]. In fact, Dorsch et al. [8], analyzed the chemical composition of leaves and stems of *Galphimia glauca* by quantitative assays and determined that 1 g of dried methanolic extract contains 6 mg of gallic acid, 12 mg methyl gallate and 125 mg of tetragalloylquinic acid. Tetragalloylquinic acid is considered a very strong antioxidant which is highly related to some of the biological activities attributed to *Galphimia glauca* [30]. 

Flavonoids are very important phytochemicals, recognized mainly for their contribution to the flavor and color of fruits and vegetables. They are distributed in different plant parts such as fruits, leaves, flowers, stems, roots, and seeds [31] and are normally ingested directly from fruits, meals, tea and wine [32]. However, it is their widely studied antioxidant properties and their positive health effects such as anti-inflammatory, anti-carcinogenic and neuroprotective that accounts for their importance. Among them, the flavonol group is the most abundant in Nature, specifically quercetin which is the most characteristic of this group [33]. This flavonoid acts as a potent scavenger of reactive oxygen and nitrogen species (ROS and RNS), superoxide and hydroxyl radicals, nitric oxide, and peroxynitrite. It can also quench free radicals and lipid peroxides [31]. Quercetin is considered a dietary flavonoid due to its wide distribution among plant foods [34]. Accordingly, some reports indicate the presence of quercetin in the methanolic and ethyl acetate extracts of *Galphimia glauca* leaves; however, the exact concentration was not described [8,29,35]. Additionally, a synergic role for quercetin along with various polyphenols against asthma and bronchial reactions to allergens have been proposed [8]. 

### 4.2. Terpenoids

Secondary metabolites comprise a large group of diverse molecules, which include the terpenoids. These chemicals are derived from the isoprene (C_5_) units that are arranged in a “head to tail” or “tail to head” fashion. They can be classified in: hemiterpenes (C_5_), monoterpenes (C_10_), sesquiterpenes (C_15_), diterpenes (C_20_), sesterpenes (C_25_), triterpenes (C_30_), tetraterpenes (C_40_) and polyterpenes (more than C_40_) [36]. Terpenoids exert different biological activities which include anticancer, analgesic, antiinflammatory, antimicrobial, antifungal, antiviral and antiparasitic [37]. 

**Figure 4:**
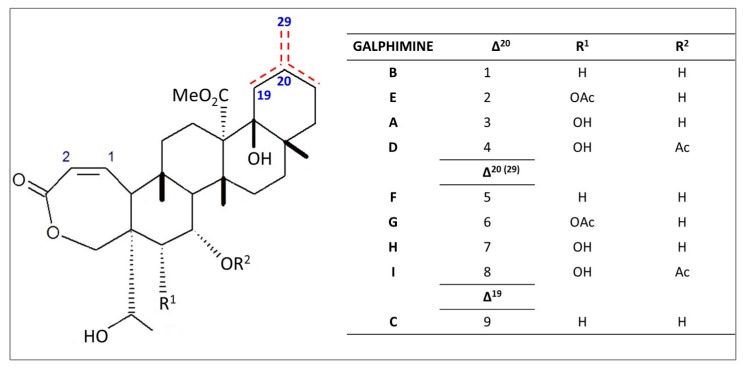
List of galphimines A–I identified in *Galphimia glauca.*

*Galphimia glauca* contains various compounds belonging to the terpenoid division of polyphenols, that mainly include “nor-seco-triterpenes,” which in this case are named the galphimine series (**1**–**9**) [4] (Figure 4). The first report of the presence of such compounds dates back to 1998 [7] when the galphimine B was discovered. Moreover, the isolation of galphimines A, B, D and the exocyclic forms for galphimines F to I has been reported [38], along with galphimine J [39]. Additionally, one of the galphimines detected, galphimine C was found to be a double bond isomer of galphimines B and F, being the only galphimine with a double bond at C-19 and C-20. Galphimine B is the major sedative compound in *Galphimia glauca* [38]. Other nor-seco-triterpenoids reported in *Galphimia glauca* include galphins A, B, and C along with galphimidin [40]. However, the most studied galphimines include galphimine B [7,41] galphimine A and galphimine E [42,43]. 

Interestingly, the content of galphimines in *Galphimia glauca* leaves can vary depending on their natural distribution as was demonstrated in a study where two main locations were tested: Dr. Mora, Guanajuato, and Jalpan de Serra, Queretaro. Thus, the major galphimine concentration was found in Dr. Mora derived plants, with a mean value for total galphimines of 6.58 mg/g DW leaves [4,44]. Furthermore, new triterpenoids glaucacetalins A–C (**1**–**3**) were reported in transformed roots [45] and gauccacetalin D in transformed cell suspension cultures of *Galphimia glauca* [46].

## 5. Pharmacological Activities 

### 5.1. Anti-Asthmatic and Anti-Allergenic Activities

Studies on active components of extracts from *Galphimia glauca* and their mechanism of action were first published in 1992. Accordingly, the potential use of *Galphimia glauca* methanolic extract in the treatment of acute bronchial reactions to allergens, and its anti-asthmatic potential in guinea pigs, was demonstrated. The main compounds responsible for this effect were found to be gallic acid, methyl gallate, and quercetin which showed a significant effect at a 45 mg/kg oral dose, while tetragalloylquinic acid displayed significant effects at a 5 mg/kg oral dose. Also, when a mixture of compounds was used, a reduction in allergen and bronchial reactions was observed [8] (Table 1). 

Furthermore, a different research group reached the same observations when comparing various phenolic compounds derived from *Galphimia glauca* leaves. In this study, the methanolic and ethyl acetate extracts revealed the presence of methyl gallate and tetragalloylquinic acid, respectively. These extracts were used to analyze its effect versus bronchial hyperreactivity and allergic reactions also. The compound that exerted the major activity against these illness markers was tetragalloylquinic acid [29]. Moreover, the effect of *Galphimia glauca* extracts on patients suffering “pollinosis” or “hay fever” has been evaluated with promising results; however, the exact mechanism and the chemical compounds associated with this effect were not reported [23] (Table 1). Also, the effectiveness of the ethyl acetate extract of *Galphimia glauca* in treating asthma-related symptoms has been analyzed. In this sense, male guinea pigs were treated with various concentrations of the extract and subjected to leukotriene (LTD_4_) induced bronchoconstriction. As a result, the *Galphimia glauca* extract was capable of reducing these symptoms in a similar way than the one reported for other chemicals that block leukotriene (LTD_4_) activity [47]. The effect of flavonoids from the aerial parts of *Galphimia glauca* in the reduction of complement induced hemolysis has been described. In this sense, isoquercetin hyperoside, gallic acid, and ellagic acid were the main compounds related to the hemolysis inhibition activity (Table 1) [35].

### 5.2. Anti-Depressive and Anxiolytic Effects 

Depression and anxiety disorders are present in 4.4% and 3.6% of the world population, respectively. These mental conditions are more prevalent in females than in males [48]. Medical treatments for these disorders rely on a plethora of pharmaceuticals which ameliorate the conditions but have a long recorded history of negative side effects [49]. Nowadays, the search for therapeutic phytochemicals is seen as a promising alternative [49,50].

#### 5.2.1. Anxiolytic and Other Effects on the CNS Evaluated in Animal Models

*Galphimia glauca* is the most studied species for the treatment of anxiety disorders in Mexico [20,44]. Accordingly, the methanolic extract derived from the aerial parts of the plant was used to evaluate the anxiolytic effect in mice through a neuro-pharmacological test [51] (Table 2). In a further study, this effect was found to be caused by the presence of galphimine B [52]. Additionally, further analysis revealed that this nor-secotriterpene compound could be exerting this anxiolytic effect due to an inhibition in dopaminergic activity [7,41] along with possible interactions with the serotonergic system [53] (Table 2).

Furthermore, the effect of galphimines A and E on anxiety was also evaluated; however, their action is less potent than that of galphimine B. Nevertheless, a study was conducted to analyze the effect of chronic administration of three main types of *Galphimia glauca* extracts in mice: aqueous, ethanolic, and methanolic. All extracts were standardized in the content of the tree main galphimines. In brief, after 56 days of administration, no changes in liver function biochemical parameters were found, and none of the three extracts was found to be genotoxic. In fact, no deaths were caused by the use of these extracts, nor any histopathological symptoms were registered [54] (Table 2). 

Since galphimines have proven activity on the CNS, a study conducted in 2009 described its effects on treating insomnia in mice. When comparing different Mexican plants used in traditional medicine for insomnia treatment, the most active extract was the methanolic extract of *Galphimia glauca*, where the effective dose was registered as 22.06 mg/kg. Also, this ED_50_ was slightly lower than the one registered for the *Cympobogon citratus* hexane extract, which is known as “té de limón” and is used in Mexican traditional medicine for insomnia treatment [55] (Table 2).

The capacity of galphimine A, one of the most abundant triterpenoids with anxiolytic activity found in *Galphimia glauca*, to act on the CNS has also been described [56]. In this study conducted in ICR Male mice, 200 mg/kg of galphimine A were used to analyze the pharmacokinetics of this triterpenoid. Since most studies over the years have been focused on the action and anxiolytic effects of galphimine B, the novelty of this study was to understand if galphimine A, which also has anxiolytic properties is capable of reaching the CNS and address its pharmacological target [56]. Chemical conversion of galphimine E to A was also conducted to give extra use to galphimine E since this compound is more abundant, but it is also an inactive triterpene. By making this chemical change, they could harness this anxiolytic compound. 

Recently, Garige et al. [57] reported the depressant effects of a stem methanol extract from *Galphimia glauca* during an in vivo study. Accordingly, Swiss albino mice were subjected to a different set of behavior analysis, all of them with the aim to elucidate the effects of the methanolic extract on CNS. In general, the authors propose that the *Galphimia glauca* extract exerts depressant effects in a similar way as with diazepam which is a drug normally used in patients suffering from anxiety, panic disorder, GAD, phobia disorder, obsessive-compulsive disorder (OCD) among others [58]. The mechanism for this drug is based in its capacity to bind to molecular subunits (α and γ) of GABA_A_ (gamma amino butyric acid type A) receptors located in the neuronal membranes of the central nervous system, which allows the opening of chloride ion channels. During all the analysis, the *Galphimia*-treated animals developed behaviors which were very similar to the ones exerted when diazepam is used.

Furthermore, *Galphimia glauca* methanolic extract can induce a delay in the onset of seizures induced by picrotoxin and pentylenetetrazole (PTZ) as well as to eliminate the deaths of animals due to convulsions. Also, a reduction of anxiety observed in the behavior and locomotor activity tests. Finally, muscle relaxing effects were detected in the plant extract treated mice. Therefore, the results of this study reinforce the role of *Galphimia glauca* in the treatment of anxiety-related disorders and the generation of calming effects on the nerves [57] (Table 2). 

In recent findings, the use of either the methanolic extract of *Galphimia glauca*, a galphimine rich fraction (GRF) or purified galphimines (G-A, G-B, and G-E) in the behavior of mice was analyzed. In this study, different extracts were evaluated for their effects on acute symptoms associated with the onset of schizophrenia, induced by ketamine in mice using the Haloperidol-induced catalepsy. Furthermore, the methanolic extract and the GRF showed positive results in treating the symptoms associated with psychosis [59].

#### 5.2.2. Human Clinical Trials for Anxiolytic Effects 

Medicinal herbal products have been designed using *Galphimia glauca* extracts. In 2007, the use of the capsules containing 310 mg of dried aqueous extract of *Galphimia glauca* (0.348 mg of galphimine B) in comparison to lorazepam in patients with a generalized anxiety disorder (GAD) was evaluated [49]. Patients were evaluated with the use of the Hamilton Anxiety Rating Scale (HAM-A) also other symptoms such as tolerability, the absence of excessive sedation and safety (absence of pathological alterations in renal and hepatic functions) were evaluated (Table 3). In conclusion, the effectiveness of this plant versus anxiety was demonstrated, and its behavior was similar to the one observed when lorazepam is used. The major difference between the use of lorazepam and *Galphimia glauca* (galphimine B) relied on tolerability since lorazepam induced excessive sedation than the plant extract. Furthermore, the anxiolytic effect caused by *Galphimia glauca* was noticed during the first week of administration, which is not the case for other drugs (azapirones and selective serotonin reuptake inhibitors, SSRIs) that require at least 3 to 4 weeks to start with this effect, therefore this is an advantage for patients that require a rapid sensation of wellbeing with a concomitant reduction of anxiety levels [49].

In a further study, the effect of a standardized dose of 0.175 mg of galphimine B in a formulation during a 15 weeks clinical trial was evaluated in patients with GAD (double-blind study) [61]. The results showed the greater effectiveness of *Galphimia glauca* over lorazepam (Table 3). The maximum anxiolytic effect was observed after the first week of administration for both groups (*Galphimia glauca* and lorazepam). However, the GB extract maintained this effect even after four weeks of administration. Furthermore, in the last weeks of the study, the plant extract exceeded the therapeutic effectiveness versus the benzodiazepine, with an overall anxiolytic effectiveness >90%. In addition, the use of GB extract was found to be better than other types of anxiolytics, namely: tricyclic antidepressants and buspirone regarding the anxiolytic effect and these pharmaceuticals started showing anxiolytic activity after the third week of administration. Finally, the use of the phytopharmaceutical presented fewer side effects than the use of lorazepam (i.e., disabling somnolence) and did not generate tolerance, intoxication, dependence or suppression syndrome during its use [61].

Recently, the effect of galphimine B in the treatment of social anxiety in young individuals was also evaluated (Table 3) [62]. In this study, purified galphimine B was administered to a group of patients from both sexes with ages ranging from 18 to 35 years old for 10 weeks, while sertraline was administered to the control group. In general, the effect of galphimine B was not significantly different from using sertraline and patients experienced a significant reduction in anxiety according to the value obtained from the Brief Social Phobia Scale (BSPS) demonstrating efficacy and safety in patients suffering from social anxiety disorder [62].

### 5.3. Antimicrobial Activity

Antimicrobial compounds in plants are normally produced for protection and defense purposes, some of them occur in the pre-infectious while others are produced as a post-infection defense system. Some of these compounds are also a product of the plant’s response to biotic stress conditions. Currently, food industry applications for natural sources of antimicrobials such as the ones found in plants is increasing [63]. In this sense, studies regarding the antimicrobial activity of plants belonging to the *Galphimia* genus are scarce. 

The first report regarding the antimicrobial potential of this plant was described in 2002, where the antiprotozoal activity of *Galphimia glauca* was evaluated [40]. Thus, the *n*-butanol and chloroform fractions of *Galphimia glauca* were found to exert moderate antiprotozoal activity against *Plasmodium falciparum* K1, *Trypanosoma brucei brucei*, *Leishmania donovani*. These fractions contained galphins A, B and C, galphimidin, quercetin, stigmasterol, and sitosterol 3-*O*-β-d-glucoside which could correlate with the activities displayed against protozoans. Nevertheless, the authors explained that quercetin exhibited a weak activity versus *plasmodium*, *trypanosoma*, and *leishmania* when compared to the standard drugs. Finally, stigmasterol and sitosterol 3-*O*-β-d-glucoside were not effective versus any of the microorganisms analyzed. 

In 2003, García et al. [64] analyzed the antifungal activities of nine Mexican medicinal plants, including *Galphimia glauca.* Plant extracts were obtained through sequential extraction with n-hexane and methanol. The action of these extracts was analyzed against diverse microorganisms such as *Aspergillus niger*, *Trichophyton mentagrophytes*, *Trichophyton rubrum*, and *Candida albicans*. Even though the minimum inhibitory concentration (MIC) of *Galphimia glauca* was not amongst the most effective plants analyzed in this study, it did show inhibition versus all fungal microorganisms analyzed, and the MIC values were above 8 mg/mL except for *Trichophyton rubrum* (4.0 mg/mL). Therefore, these activities can rely on the plethora of phytochemical compounds found in these plant species, namely flavonoids and terpenes, among others [64].

Other studies focused on the *Galphimia* genus include the analysis of antioxidant, brine shrimp lethality and antimicrobial activity in *Galphimia gracilis* Bartl. in 2015 [65]. The antimicrobial activity was evaluated against *Bacillus subtilis*, *Staphylococcus aureus*, *Escherichia coli*, *Salmonella typhi*, *Salmonella abony*, and *Pseudomonas aeruginosa*. Furthermore, three different extracts were utilized: methanol, ethyl acetate, and petroleum ether. The results of the antimicrobial activity analysis revealed that the methanol extract was more effective against *E. coli*, *S. typhi*, and *P. aeruginosa*, while the ethyl acetate extract was more effective against *S*. *typhi* and *P*. *aeruginosa*. Ethyl acetate extract exhibited the highest antimicrobial activity. However, the authors consider that all the extracts showed poor activity when compared to the control (azithromycin). 

### 5.4. Antiinflammatory Activity

As stated previously, *Galphimia glauca* has been utilized since pre-Hispanic times to treat diverse illness which includes gastric ulcers, traumatic blows, wounds, scars, kidney, and uterus inflammation as well as rheumatism. All these medical conditions have the inflammatory process in common. In addition, this plant contains a high amount of polyphenol compounds that can address these conditions and serve for their treatment. 

As part of a major study, an antiinflammatory test was done in mice using the methanolic extract of different cultivars of *Galphimia glauca*. As a result, this plant extract was capable of reducing the inflammation in mouse ears which was previously induced by tetradecanoyl phorbol acetate [44]. Furthermore, González-Cortázar et al. [60] analyzed the antiinflammatory properties of *Galphimia glauca* crude extracts. To do this, n-hexane, ethyl acetate, dichloromethane, and methanol extracts were analyzed. This study revealed that the galphimine A and galphimine E were the main anti-inflammatory principles present in the methanolic extract [60] (Table 2). 

### 5.5. Antiproliferative Activity 

The use of plants as main sources of chemical compounds with antiinflammatory, antiproliferative or cytotoxic potential has long been reported. This constitutes the basic studies when linking a particular set of compounds or fractions and its effects on in vitro analysis with their potential health benefits. [66]. In some cases, these results are applied by the phytopharmaceutical or biopharmaceutical industries in their formulations.

In the particular case of *Galphimia glauca*, a report indicates the evaluation of different extracts versus several cancer cell lines. In 2007, Aguilar-Santamaria et al. [54] analyzed the toxicological effects of *Galphimia glauca* aqueous, methanolic and ethanolic extracts regarding its effects on different parameters. For the hepatoxicity analysis, the enzymatic profiles of alkaline phosphatase (ALP), alanine aminotransferase (ALT) and aspartate aminotransferase (AST) were evaluated. The results showed no hepatotoxic effects of these extracts in mice. Also, they developed cytotoxicity studies in different cell lines such as HCT-15 (colon), UISO (uterus), KB (nasopharyngeal) and OVCAR-5 (ovarian cancer); as a result, all the extracts analyzed exhibited an ED_50_ higher than 20 µg/mL on nasopharyngeal, UISO and OVCAR-5 cell lines. However, a cytotoxic effect in the colon cell line was found, where the ED_50_ for all extracts was lower than 2 µg/mL. Finally, the genotoxicity analysis revealed that the evaluated plant extracts (50, 100, and 250 µg/mL) had no genotoxic effects. Therefore, the authors suggest that the use of *Galphimia glauca* extracts possess very low risks of toxicity and genotoxicity that is in line with the therapeutic safety guidelines for phytopharmaceuticals [54].

### 5.6. Vasoactive Effect and Spasmolytic Activity 

The infusions of flowers of *Galphimia glauca* to treat heart pain has also been reported in the traditional medicine system of Mexico [67]. In this sense, a study regarding the potential use of *Galphimia glauca* as source of compounds with vasoactive effect has been reported [68]. An aqueous extract was prepared from leaves and flowers of *Galphimia glauca* which was evaluated in thoracic aorta from adult male Wistar rats. This analysis revealed that leaves extract of *Galphimia glauca* was more active than the flowers extract in a concentration dependent manner. 

Other traditional uses of *Galphimia glauca* indicate its application for the treatment of stomach related problems [6], in this sense the methanolic extract of *Galphimia glauca*, which is known for its sedative properties was used to evaluate its spasmolytic effect in the ileum of guinea pigs [39]. Accordingly, several fractions were tested to evaluate their capacity to reduced contractions that were induced artificially. As a result, the use of the methanolic extract reduced the contractions by 80.42%. Among all the fractions evaluated, galphimine F showed four times higher inhibition than galphimine B. 

## 6. Toxicity Tests

The use of plants in the formulation of phytopharmaceuticals is increasing day by day. Due to the importance of this plant species in the treatment of mental disorders, such as anxiety the analysis of toxicological effects is relevant. 

To the best of our knowledge, the Lethal Dosage (LD_50_) for *Galphimia glauca* has not been determined yet. A closely related species *Galphimia gracilis* was evaluated with brine shrimp (*Artemia salina* Leach) through bioassay analysis [65]. In this study, three different plant extracts were used: methanol, ethyl acetate, and petroleum ether. Among them, methanol extract was found to be the most toxic to brine shrimp nauplii with a LC_50_ of 64.46 µg/mL, while the ethyl acetate extract exhibited a LD_50_ of 131.88 µg/mL, and the petroleum ether extracts a LD_50_ of 225.42 µg/mL. According to the authors, LD_50_ values below 500 µg/mL are considered toxic [69]. Therefore, the results observed for the different *Galphimia gracilis* extracts are promising versus further studies involving cancer cell lines. Furthermore, the observed LD_50_ values can easily correlate with the different types of polyphenolic compounds found within the *Galphimia* genus [65]. 

## 7. Biotechnological Studies 

The use of phytochemical compounds from a natural origin is tightly linked to human civilization and development. Many of these compounds present in plants drive the increasing demand for pharmaceuticals, without them more than half of the drugs from which we benefit today might not have existed [70]. 

The use of biotechnology to improve yields of medicinal plants such as in the case of plant tissue culture is a promising tool to avoid germoplasm extinction. Therefore, the first studies related to a tissue culture of *Galphimia glauca* reported in 1999 [71]. The authors described a methodology for callus formation, where the best callus induction medium consisted of Murashige and Skoog (MS) supplemented with 2 m/L of 2,4-dichlorophenoxyacetic acid (2,4-D). The cellular growth of these calli was observed when 2 mg/L naphthaleneacetic acid (NAA) plus 1 mg/L kinetin (KN) were employed. The subculture of these callus structures in 2,4-D (4 mg/L) medium resulted in increased production of galphimine B and 6-acetoxygalphimine B [71].

In a further study, this same research group described the in vitro production of galphimine B in cell suspension cultures [72]. Thus, a cell suspension batch culture was employed, and the effect of the main parameters (inoculum size, plant growth regulators and various sucrose, nitrate, and phosphate concentrations) were analyzed. As a result, a major cellular growth was found when 2 mg/L NAA + 1 mg/L KN was utilized. Finally, when 4 mg/L 2,4-D was used a marked increase in galphimine B accumulation was observed (36% higher concerning calli, and wild plants).

Also, Rojas et al. [73] developed a micropropagation procedure for *Galphimia glauca* and the establishment of plantlets in the field. In addition, they also evaluated the contents of galphimine B in both wild and micropropagated plants. Various concentrations of both IAA and KN were evaluated. However, the best treatment for shoot induction was found to be MS supplemented with 4 mg/L KN. Still, the combination of 4 mg/L KN + 0.1 mg/L IAA produced the highest shoot length and the highest pair of leaves per explant. The formation of roots was observed 20 days after ex vitro indol butyric acid application (IBA) and is consequent transplantation to soil conditions. The plantlets were transferred to greenhouse conditions for two months, and after this period they were moved to field conditions, where they exhibited a 90% survival with the first flowering event taking place only four months after they were transplanted. The content of galphimine B in methanol extracts from either, wild and micropropagated plants was very similar (7.83 and 7.39 mg/g DW, respectively). In brief, the authors suggest that the use of this micropropagation technique can be used for the conservation of this important medicinal plant [73]. 

Since galphimines are very important compounds to achieve the anxiolytic effect in *Galphimia glauca*, experiments using the in vitro tissue culture techniques to increase their yield and production have also been done. A cell suspensions culture for *Galphimia glauca* were developed specifically for galphimine B production [74]. In this study, a two-stage culture system was used, first immobilization in Ca^2+^ alginate beads and scale up from flask to bioreactors (stirred and airlift). As a result, cells immobilized in the matrix excreted up to 100% of the galphimine B produced. In this study a stirred type bioreactor showed promising results, generating galphimine B content up to 1381 µg/L after 24 days of culture.

Plant genetic transformation has also been achieved for *Galphimia glauca*. A report describing the production of galphimines using *Agrobacterium rhizogenes* was described by [45]. The transformation process was done on cotyledons and hypocotyls of *Galphimia glauca* seedlings grown in Gamborg B5 solid medium devoid of plant growth regulators. Then, the explants were transferred into a new medium containing 200 mg/L timentin and 2 g/L polyvinylpyrrolidone (PVP) for hairy root induction. The main objective of this research, besides generating a genetic transformation protocol, was to assess the production of nor-friedelanes via hairy root production. Hence, the hairy root nutrient medium was used to analyze the yield of these compounds. The transformed roots generated three major nor-friedelanes, namely glaucacetalins A–C that were found in the nutrient medium [45]. 

The same research group described the production of triterpenoids in liquid cultivated hairy roots of “*calderona amarilla*” [75]. Thus, galphimine E, glaucacetalin A, and mascalinic acid were obtained from this process. Glaucacetalin A was excreted to the culture media presenting a maximum concentration of 2.14 mg/L after 21 days of culture. On the other hand, galphimine E and mascalinic acid were obtained in the root biomass with concentrations as high as 0.11 and 0.43 mg/g, respectively after 39 days in culture [75]. Another study involving cell suspension cultures from *Galphimia glauca* (GgBa) led to the discovery of a novel triterpenoid compound related with galphimines: glaucacetalin D with an overall yield of 2.9 mg/L. The reported glaucacetalin D exerted a sedative effect similar to galphimine B [46]. 

Plants respond to abiotic or biotic stress by inducing the expression of certain metabolic pathways, most of them are also related with the production of secondary metabolites. Jasmonic acid or methyl jasmonate [the methyl ester form (MeJA)] are part of this defense response in plants [76]. The role for these compounds in the induction of triterpenes and sterols has been analyzed in several plants, including *Galphimia glauca* [77]. Thus, the effect of 100 µM MeJA on roots of *Galphimia glauca* was evaluated. Root growth inhibition was observed, which resulted in plantlet premature death. However, the exposure of roots to MeJA led to the increase in galphimine B production (≈17 µg/g DW) as well as the cholesterol levels (15% of all total free sterol content). This constitutes the first report regarding the use of elicitor molecules in the nor-seco friedelane galphimine metabolic pathway [77]. 

## 8. Taxonomical Misinterpretations in the *Galphimia* Genus

Showy inflorescences and morphological similarity have led to the incorrect labeling of the species of this genus which was formerly known as *Thryallis* L, now, *Galphimia.* Also, the morphological resemblance among various members of the *Galphimia* genus has misled collectors to incorrectly assign names for the specimens collected, mainly labeled as *Galphimia glauca* and *Galphimia gracilis* [3]. 

Further studies have demonstrated disagreements at the metabolomic and genetic level between plants allegedly classified as *Galphimia glauca* in various regions of Mexico. Accordingly, a study discovered that only certain regions of Mexico produce *Galphimia glauca* specimens that contain high levels of “galphimines” which are responsible for its anxiolytic and sedative effects, as well as other important medicinal properties. This was confirmed through a metabolomics analysis done in plants collected in different localities of Central Mexico. This analysis revealed that plants collected from only two regions (Dr. Mora, Guanajuato, and Jalpan, Queretaro) were capable of producing galphimines, while the others fail to do so, or produced them at a considerable lower rate [3]. 

On a further study, Sharma et al. [44] analyzed four years later the metabolomic profile of these same localities plus two new ones and found that populations collected from Dr. Mora and Jalpan were the only ones that produce galphimines and labeled them as active after evaluating them through mouse models to test anxiety and sedative effects. These results suggest that the galphimine production in these plants can be related to environmental factors that could help increase or decrease their production, in fact, most of the plants analyzed grow in contrasting environments. 

A second study utilizing DNA barcoding as a molecular marker confirmed the clear differences among the plant populations used in the former study [78]. The results found in this study indicated that the plants from all seven populations analyzed do not belong to the one *Galphimia glauca* species as labeled in the respective herbarium, on the contrary, there could be at least three different species of the genus *Galphimia*. Also, TLC results were consistent with the expected galphimine production profile in the two main “producing” populations: Dr. Mora Guanajuato, and Jalpan Queretaro.

## 9. Patents

In 2007, Tortoriello et al. registered a patent for the dry extract production from *Galphimia glauca* and use of a pharmaceutical composition in the treatment of anxiety [79]. Accordingly, the authors described that their invention is related mainly with the pharmaceutical formulation comprising a dry extract from “*calderona amarilla*” which is obtained after various solvent extraction procedures. Thus, the formulation generated is to be used for treatment, prevention and possible cure of anxiety in all its forms. To the best of our knowledge, this is the only patent existing for *Galphimia glauca* extracts until now. 

## 10. Current Needs

The present data demonstrate the pharmacological potential of *Galphimia glauca* to treat mental disorders such as anxiety, insomnia, and depression. Millions of people worldwide suffer from mental problems, and the treatment of these ailments is restricted to some synthetic drugs which induce from mild to severe side effects, including dependence. *Galphimia glauca* has shown comparatively superior medicinal properties, but its uses are still limited to traditional preparation. Development of phytomedicines with standardized extracts would be of great support for the treatment of patients with such a disorder. The available data on *Galphimia glauca* represents a wide range of traditional as well as potentially new health applications especially anti-inflammatory, cytotoxic and antimalarial properties. Limited data that correlates the traditional/therapeutics uses of *Galphimia glauca* and phytochemical profile of the extract used are available.

Most of the literature used to prepare this review is mainly focused on the type of extract and part of the plant but, do not identify or quantify bioactive compounds. Modern metabolomics tools could be very useful to study and identify novel compounds and to develop a complete phytochemical profile. The functions of galphimines are not known in the plant, so further research is required to understand their importance in nature. Exploring their role in the plant may open new research areas. Special emphasis is needed in the correct identification of the plant material because of many closely related species of the genus share very similar morphological appearances which disoriented collector, taxonomist, and researcher. Scientists inside or outside of Mexico should identify their plant material of *Galphimia glauca* or related species as reported by the authors [3,69] for reliable scientific production, so DNA barcoding using *matK* and *rpoc1*, as well as TLC analysis could be useful for the correct identification to develop authentic research data or even a reliable final product. The natural habitat for *Galphimia glauca* is limited to several states of Mexican territories such as Aguascalientes, Guanajuato, Hidalgo, Jalisco, Nuevo Leon, Querétaro, Tamaulipas, San Luis Potosí and Zacatecas but most of the research data presented in this review have utilized the samples collected from Guanajuato and Queretaro. Local researchers should identify and declare the new localities within the Mexican territory. In our own experience, the collection of this plant species is not an easy task because very little or no information is available about its geographical distribution. Many reported sites in the literature are too old and have been converted into agricultural farms or urban areas. The local herbal market is depending on the availability of the plant material in restricted locations, and it is completely sessional. There is very little, or no information available on the plant material collected from other states. Constant collection of the plant material from its natural habitat may put the species on the risk of overexploitation. More studies with a focus on conservation and agronomical practices are highly required. Micropropagation has been reported, but its scientific or commercial exploitation is not known. More studies to develop fast and scale-up micropropagation protocols will be highly helpful to produce the plant for research and commercial purposes. To be able to develop phytomedicine or synthetic drug for the treatment of CNS disorders, multidisciplinary kind of research on *Galphimia glauca* is highly required. Genomics and transcriptomics are the promising areas that can help in understanding the biosynthesis pathways that lead the production of important triterpenoid family. Bioreactor-based investigations have been reported, but no significant advances are achieved for the further scale-up production of desired galphimines. 

## 11. Conclusions

Plants of the genus *Galphimia* are mainly distributed throughout Mexico, and some species have been extended to Texas, USA and Central America including Guatemala and Brazil. In this review, we have emphasized on the species *Galphimia glauca* because most of the research has been centralized on this species especially that is correlated with the therapeutic uses for the CNS disorders. With regards to 26 species of the genus, there are many species that have received very little of no attention. In this review, we have condensed the biological activities and reported bioactive compounds that represent most of the scientific literature published on *Galphimia glauca*. The present information helps in filling the gap between scientific studies and traditional use of this plant. The present review also suggests the current needs of multidisciplinary research such as agronomical practices, tissue culture protocols, phytochemical studies, bioactive compound identification, bioreactor scale-up studies, recognitions of genes involved in the biosynthesis, and molecular taxonomy for *Galphimia glauca*. 

## Figures and Tables

**Table 1:** Phytochemical compounds with beneficial health effects found in *Galphimia glauca.*

Type of Compound	Compound	Plant Part Used	Extract	Dose	Model	Effect	Reference
**Phenolic**	Gallic acid Methyl gallate Tetragalloyl quinic acid	Leaves and stems	Methanolic	2 mg/kg body weight	Guinea pigs	Inhibition of acute bronchial reactions	[8]
	Tetragalloyl quinic acid Gallic acid Methylgallate Ellagic acid	Aerial parts	Ethanolic	5 mg/kg body weight	Guinea pigs	Bronchial hyperreactivity and allergic reactions	[29]
	Tetragalloyl quinic acid	Aerial parts	Ethyl acetate	100 µg/mL	Guinea pigs	Asthma-related symptoms	[47]
**Flavonoids**	Quercetin	Leaves and stems	Methanolic	2 mg/kg body weight	Guinea pigs	Inhibition of acute bronchial reactions	[8]
	Quercetin	Aerial parts	Ethyl acetate	5 mg/kg body weight	Guinea pigs	Bronchial hyperreactivity and allergic reactions	[29]
	Quercetin 3-*O*-(2″-galloyl)-β-d-glucoside Quercetin-3-*O*-(6″-galloyl-)-β-d-glucoside	Aerial parts	Ethyl acetate	100 µg/mL	Guinea pigs	Reduction of complement induced hemolysis	[35]

**Table 2:** Pharmacological effects of galphimines and related compounds from *Galphimia glauca* tested in vivo in animal models.

Compound	Extract	Plant Part Used	Model	Analysis	Disorder	Dosage	Controls	Result	Ref.
Galphimine B	Crystallized galphimine B	Aerial parts	79 male Wistar rats.	Effects on cerebral activity	Central nervous system diseases	0.5, 1.0 and 2.5 mg/kg	10% polyethyleneglycol	Administration (systemic and localized) of Galphimine B, demonstrated excitatory effects in neurons localized mainly in the Ventral Tegmental Neurons that is a target for antipsychotic drugs	[7]
Galphimine B	Purified Galphimine B	Aerial parts	Wistar rats	Effects on VTA neurons through patch clamps		1 μM–5 mM	Not reported	Action upon dopaminergic VTA neurons in a nonGABAergic mechanism	[41]
Galphimines A, B, and E	Fractionation of the methanolic extract	Aerial parts	Male ICR mice	Elevated Plus Maze	Anxiety	15 mg/kg of purified galphimines or Galphimine Rich Fraction (GRF)	5% Tween 20	Anxiolytic effect induced not significant differences with diazepam	[42]
Galphimines	Methanolic	Aerial parts	ICR albino mice	Open arms in elevated plus maze, light dark paradigm test, forced swimming test	Anxiety and depression	125, 250, 500 and 2000 mg/kg	Not reported	Anxiolytic like effect	[43]
Galphimines	Ethylacetate	Aerial parts	Male guinea pigs	Leukotriene D4 (LTD4) induced bronchoconstriction	Asthma	10, 31.6, 56.2 and 100 μg/mL	Not reported	Similar than SK&F 104353 an LTD4 antagonist.	[47]
Galphimines	Methanolic	Aerial parts	10 Wistar Rats	Strictine induced convulsions	Convulsions	1, 10, 50, and 100 mg/b g of methanolic extract	10% Tween 80	Decrease in seizures and reduction in mortality at the 50 mg/100 g dose	[51]
Galphimines	Methanolic	Aerial parts	10 Albino mice	Protection against leptazol-induced convulsions	Convulsions	50 mg/I 00 g i.p.	10% Tween 80	Decrease in seizures and reduction in mortality at the 50 mg/100 g dose	[51]
Galphimines	Methanolic	Aerial parts	10 Male albino mice	Barbiturate potentation	Convulsions	1, 10, 50 mg/b g	10% Tween 80	Increase of sleeping time induced by sodium pentobarbital in a dose-dependent manner, higher effect at 50 mg/100 g	[51]
Galphimine B	Crystallized galphimine B	Aerial parts	Male albino mice	Strychnine-induced convulsions	Convulsions	10, 40, 80 mg/kg	10% Tween 80	Not a significant effect	[52]
Galphimine B	Crystallized galphimine B	Aerial parts	Male albino mice	Leptazol-induced convulsions	Convulsions	10, 40, 80 mg/kg	10% Tween 80	Not a significant effect	[52]
Galphimine B	Crystallized galphimine B	Aerial parts	Male albino mice	Potentiation of general anesthetics	Convulsions	10, 40, 80 mg/kg	10% Tween 80	Significant increase in narcosis time induced by sodium pentobarbital. Highest effects found at 80 mg/kg	[52]
Galphimines	Methanolic Hexane	Aerial parts	Male ICR mice	Exploratory cylinder test	Insomnia	22.06 mg/kg	0.05% Tween 80 in saline solution	Sedative effects in mice.	[55]
Galphimine A	Ethyl acetate	Aerial parts	Male ICR mice	Pharmacokinetic study	Anxiety	200 mg/kg	Not reported	Anxiolytic effect of galphimine A in the CNS	[56]
Galphimines A, B and E	Methanolic fraction, Galphimine Rich Fraction Galphimine A, B and E		Male ICR Mice	Open Field Test Passive Avoidance Test Forced Swimming Test	Behavioural changes	Metanol extract: 25, 100, 250 and 500 mg/kg p.o GRF: 5, 15, and 30 mg/kg p.o, GA, GB and GE (5, 10 and 30 mg/kg p.o	1% Tween 20	The effect caused partially by their interaction with dopaminergic and glutaminergic systems in vivo. Protection against hallucinations and psychosis.	[59]
Galphimines	*N*-hexane, ethyl acetate, dichloromethane and methanol	Leaves	Male ICR mice	Acute inflammation with TPA	Inflammation	3.2 mg/ear	Indomethacin	Antiinflammatory principles were attributed to Galphimine A and Galphimine E	[60]

**Table 3:** Human clinical trials to treat anxiety related disorders reported for *Galphimia glauca*.

Clinical Trial	Extract (Alone or in Combination)	Plant Parts Used	Dosage	Duration of the Study	Controls	Sample (Number of Patients)	Scale	Result	Tolerability, Security or LD_50_	Reference
Double blind study, randomized, lorazepam controlled	Aqueous	Leaves and stems	310 mg of dried aqueous extract capsules	4 weeks	Lorazepam	152	HAM	Same anxiolytic effect than Lorazepam. Side effects reduction	Well tolerated	[49]
Double blind study, randomized, lorazepam controlled	Purified galphimine B	Leaves and stems	0.175 mg of galphimine B 12 weeks	12 weeks	Lorazepam	191	HAM	Anxiolytic effect superior to Lorazepam	Well tolerated	[61]
Double blind randomized study	Purified galphimine B	Aerial parts	0.374 mg/dose galphimine B	10 weeks	Sertraline	34	BSPS	No significant difference with the use of sertraline	Well tolerated	[62]

HAM-A: Hamilton Anxiety Scale; BSPS: Brief Social Phobia Scale.

## Abbreviations

CNS: central nervous system; ROS: reactive oxygen species; DW: dry weight; GAD: generalized anxiety disorder; HMA: Hamilton Anxiety Rating Scale; SSRI: serotonin reuptake inhibitors; GB: galphimine B, OCD: Obsessive Compulsive Disorder; GABA: γ-aminobutyric acid type A; PTZ: pentylenetetrazole.
